# The grim reaper evading modern medicine: aspergillosis, adenovirus, and Hodgkin lymphoma

**DOI:** 10.4322/acr.2020.191

**Published:** 2020-09-02

**Authors:** Larry Nichols, Justin Armstrong, Cody Atkinson

**Affiliations:** a Mercer University School of Medicine, Department of Pathology. Macon, GA, USA.; b Mercer University, School of Medicine. Macon, GA, USA.

**Keywords:** Aspergillosis, Adenovirus, Hepatitis, Hodgkin lymphoma: Autopsy

## Abstract

Illustrative cases of diseases that are difficult to suspect and diagnose can serve as useful reminders. Invasive pulmonary aspergillosis and adenovirus hepatitis are two such diseases, both revealed by autopsy in this case of Hodgkin lymphoma refractory to chemotherapy treated with allogeneic hematopoietic stem cell transplantation complicated by these two fatal infections. This patient was cured of Hodgkin lymphoma, *Clostridioides difficile* colitis and thrombotic thrombocytopenic purpura using the marvels of modern medicine. This case illustrates many features of aspergillosis and adenovirus hepatitis, shows the value of autopsy in revealing diagnoses, and illustrates the limits of modern medicine, which should serve as a mental spur in our efforts to advance medical science, to try to defeat the numerous demons of disease, who seem to keep outwitting us.

## CASE REPORT

This 38-year-old white male had stage IV nodular sclerosing Hodgkin lymphoma first diagnosed 21 months ago. Over the following 6 months, he was treated with 2 regimens of combination chemotherapy, first with ABVD (Adriamycin [doxorubicin], bleomycin, vinblastine and dacarbazine), and then ESHAP (etoposide, Solu-Medrol [methylprednisolone], high-dose Ara-C [cytarabine] and Platinol [cisplatin]). He had a history of smoking cigarettes, 1.5 packs/day, ending 13 months ago. He received an autologous stem cell transplant 7 months ago and underwent chemotherapy with gemcitabine 4 months ago. He underwent allogeneic HLA-matched stem cell transplant 3 months ago with cyclosporine for prophylaxis against graft-versus-host disease. He developed diarrhea with positive stool assay for *Clostridioides difficile* toxin, which started 5 days after transplantation and was improved but not eliminated with oral vancomycin and metronidazole therapy. The patient’s stool became negative for C. difficile toxin. He had evidence of stem cell engraftment and was discharged from the hospital on metronidazole, acyclovir, fluconazole and pentamidine. The patient had persistent diarrhea despite oral metronidazole therapy, suggesting the possibility of graft-versus-host disease, for which oral prednisone therapy was added to his regimen 7 weeks ago. Ileal and colonic biopsies showed no abnormalities, but biopsy culture was positive for adenovirus, 6 weeks ago, treated with a brief course of cidofovir and one dose of intravenous immunoglobulin, stopped when the patient’s serum creatinine increased and his white blood cell (WBC) count decreased.

The patient developed thrombocytopenia, associated with the presence of schistocytes in his peripheral blood, high serum lactate dehydrogenase (LDH) and low haptoglobin, 1 month ago. Thrombotic thrombocytopenic purpura was diagnosed and attributed to cyclosporine; it was treated with plasmapheresis, rituximab and high-dose corticosteroid therapy, but the patient’s platelet counts remained low. He was maintained on rituximab, mycophenolate, metronidazole, fluconazole, acyclovir, pentamidine and tapering doses of prednisone.

The patient was hospitalized with dyspnea and recent fever unresponsive to adding ciprofloxacin and azithromycin to his regimen. On admission, his temperature was 37.7 °C, pulse 85/minute, blood pressure 170/100 mm Hg, respirations 18/minute and oxygen saturation 98%, on supplemental oxygen at 2 L/minute via nasal cannula. He had wheezes over right upper lobe lung, along with a distended abdomen and moderate leg edema. His WBC count was 1,900/mm^3^ (reference range [RR]: 3,800-10,600/mm^3^) (93.5% neutrophils, 3.7% monocytes, 2.8% lymphocytes), hemoglobin (Hgb) 8.7 g/dL (RR: 12.9-16.9 g/dL), platelet count 17,000/nmm^3^ (RR: 156,000-369,000/mm^3^), international normalized ratio (INR) 1.2, glucose 116 mg/dL (RR: 70-99 mg/dL), creatinine 3 mg/dL (RR: 0.5-1.4 mg/dL), albumin 2.8 g/dL (RR: 3.5-5 g/dL), total bilirubin 0.7 mg/dL (RR: 0.3-1.5 mg/dL), alkaline phosphatase (ALP) 72 U/L (RR: 40-125 U/L), gamma-glutamyl-transferase (GGT) 54 U/L (RR: <30 U/L), alanine aminotransferase (ALT) 85 U/L (RR: <40 U/L), aspartate aminotransferase (AST) 123 U/L (RR: <40 U/L), and LDH 1459 U/L (RR: <170 U/L). Computed tomography (CT) scan of the chest showed a right perihilar 4.5 x 4.3 cm infiltrate and a 2.6 x 2.3 cm rounded lateral right upper lobe infiltrate. The patient was treated with piperacillin/tazobactam, caspofungin, acyclovir, metronidazole, metoprolol, hydralazine, clonidine, furosemide, filgrastim, vincristine, mycophenolate, prednisone (40 mg/day) and methylprednisolone (50 mg/day).

On hospital day 2, the patient’ had a cough productive of blood-tinged sputum. His maximum temperature was 38.2^o^ C, pulse 72/minute, blood pressure 162/100 mm Hg, respirations 20/minute, and oxygen saturation 100% on supplemental oxygen at 4 L/minute. He had left basilar pulmonary crackles, and right upper lobe wheezes. His WBC count was 1,500/mm^3^ (84% segmented neutrophils, 6% bands), Hgb 7.2 g/dL, platelets 6,000/mm^3^, and creatinine 3.2 mg/dL.

On day 3, the patient had a cough and low-grade hemoptysis. His maximum temperature was 37.5^o^ C, pulse 88/minute, blood pressure 158/98 mm Hg, respirations 16/minute, and saturation 93% on oxygen at 3 L/minute. He had coarse pulmonary crackles half-way up his left chest posteriorly. His WBC count was 1,900/mm^3^ (85% segmented neutrophils, 8% bands), Hgb 8.8 g/dL, platelets 13,000/mm^3^, and creatinine 3.3 mg/dL. Voriconazole was added to his treatment regimen. On day 4, at night, the patient had the new onset of confusion.

On day 5, the patient had dyspnea and diarrhea (2-3/day), along with bilateral pulmonary rhonchi (especially over left upper lobe). His WBC count was 1,100/mm^3^ (92% segmented neutrophils, 6% bands), Hgb 9.1 g/dL, platelets 11,000/mm^3^, INR 2.1, creatinine 4.2 mg/dL, bilirubin 0.9 mg/dL, ALP 125 U/L, ALT 215 U/L, AST 444 U/L, and LDH 3753 U/L. Chest CT demonstrated a 5.5 x 5.3 cm right perihilar infiltrate. a 2.9 x 2.7 cm right upper lobe infiltrate, ground glass opacity throughout the right lung, new multifocal ground glass opacity in the left lung, a new 2.4 x 1.2 cm right middle lobe lung lesion, and new subtle subcentimeter densities in the liver.

On day 6, the patient suffered acute deterioration, with respiratory distress, agitation, and confusion. His pulse was 110/minute, blood pressure 160/110 mm Hg, and respirations 40/minute on oxygen via non-rebreather facemask. He was intubated and bronchoscopy revealed copious bloody bronchial secretions. His WBC count was 700/mm^3^ (92% neutrophils), Hgb 8 g/dL, platelets 70,000/mm^3^, INR 3, creatinine 5.3 mg/dL, bilirubin 1.8 mg/dL, ALP 323 U/L, ALT 337 U/L, AST 879 U/L, and LDH 8805 U/L. Posaconazole, sargramostim, and tacrolimus were added to the patient’s regimen. The patient was also treated with plasmapheresis, along with transfusion of 3 units of red blood cells and 5 units of fresh frozen plasma.

On day 7 the patient had hemoptysis, along with pulmonary rhonchi and wheezes on examination. His maximum temperature was 36.8 °C, pulse 98/minute, blood pressure 150/106 mm Hg, respirations 32/minute, and saturation 96% on ventilation with 50% oxygen and 5 cm H_2_O of positive end-expiratory pressure. His WBC count was 200/mm^3^ (68% neutrophils, 8% bands, 4% metamyelocytes, 8% myelocytes), Hgb 6.8 g/dL, platelets 13,000/mm^3^, INR 2.1, glucose 189 mg/dL, creatinine 4.3 mg/dL, albumin 2.7 g/dL, bilirubin 4.9 mg/dL, ALP 305 U/L, ALT 569 U/L, AST 2233 U/L, and LDH 18,341 U/L. He was transfused 2 units of red blood cells and 12 units of platelets. He was given vitamin K; tacrolimus and meropenem were added. Arterial blood showed pH 7.306, PCO2 33.9 mm Hg, PO2 94.3 mm Hg and bicarbonate 16.4 mEq/L (RR: 22-26 mEq/L). The patient’s respiratory rate rose to 40/minute and his heart rate increased to 110/minute. He proceeded to suffer progressive hypotension refractory to four-agent vasopressor support with norepinephrine, epinephrine, phenylephrine and vasopressin, and he died. Right upper lobe transbronchial biopsy from the day before was reported later that day showing organizing pneumonia with fibrinous exudate and negative Grocott methenamine silver stain for fungi, while bronchoalveolar lavage cytology was reported positive for fungal hyphae. The following day, blood galactomannan was reported positive at 1.8 (RR: <0.5), and four days after that, bronchoalveolar lavage respiratory viral culture was reported positive for adenovirus.

## AUTOPSY FINDINGS

**Figure 1:**
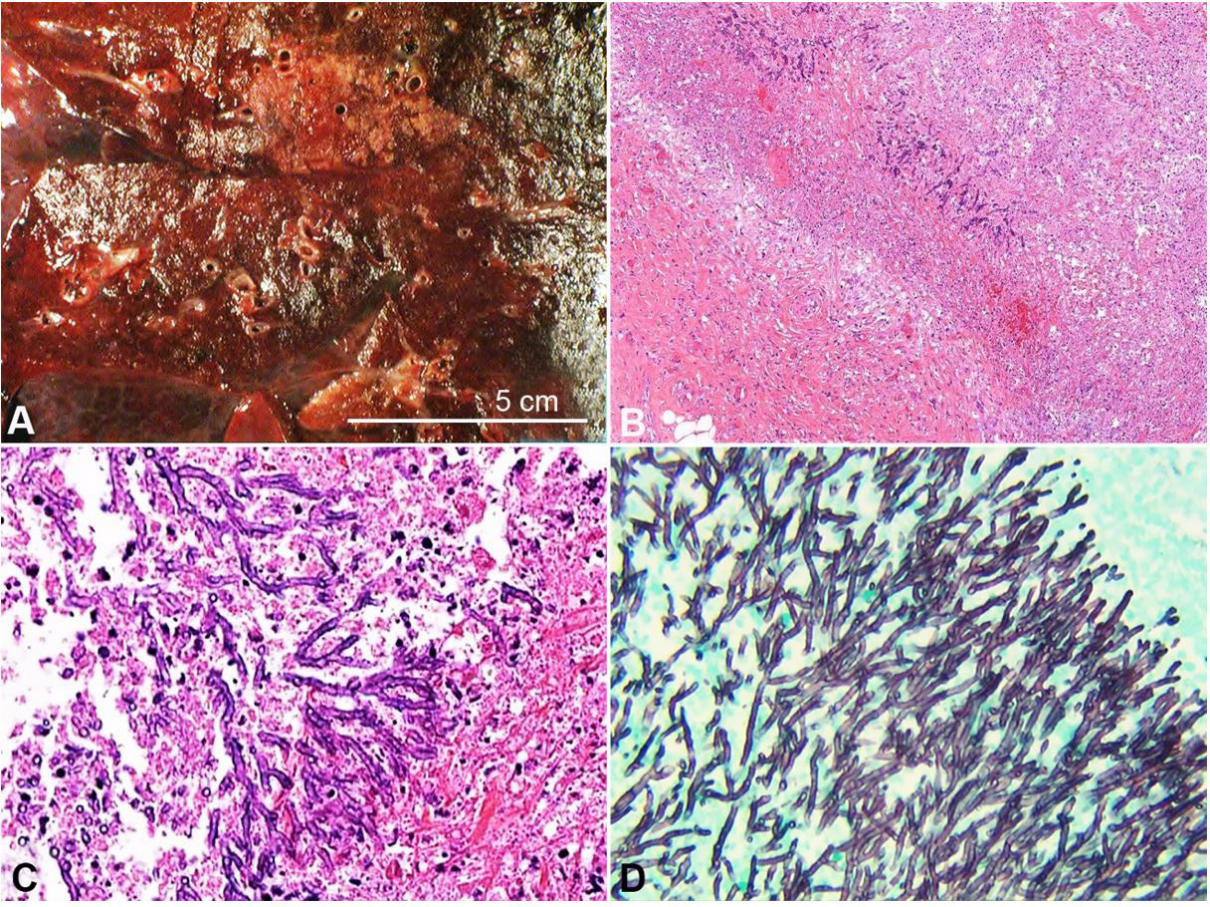
**A –** Gross view of the cut surface of lung showing diffuse severe consolidation, mostly dark red-brown, but with some lighter tan-brown areas, **B, C** and **D** photomicrographs of the lung; **B –** Edge of consolidated lung with necrotic debris, upper right, fungal hyphae and foci of hemorrhage, center, and perihilar connective tissue, lower left (H&E, 40X); **C –** Necrotic lung parenchyma with septate fungal hyphae showing progressive acute angle branching; note the loosening of the necrotic tissue on the way to forming a microabscess (H&E, 200X); **D –** Dense radial array of septate fungal hyphae with acute angle branching (GMS, 200X).

**Figure 2:**
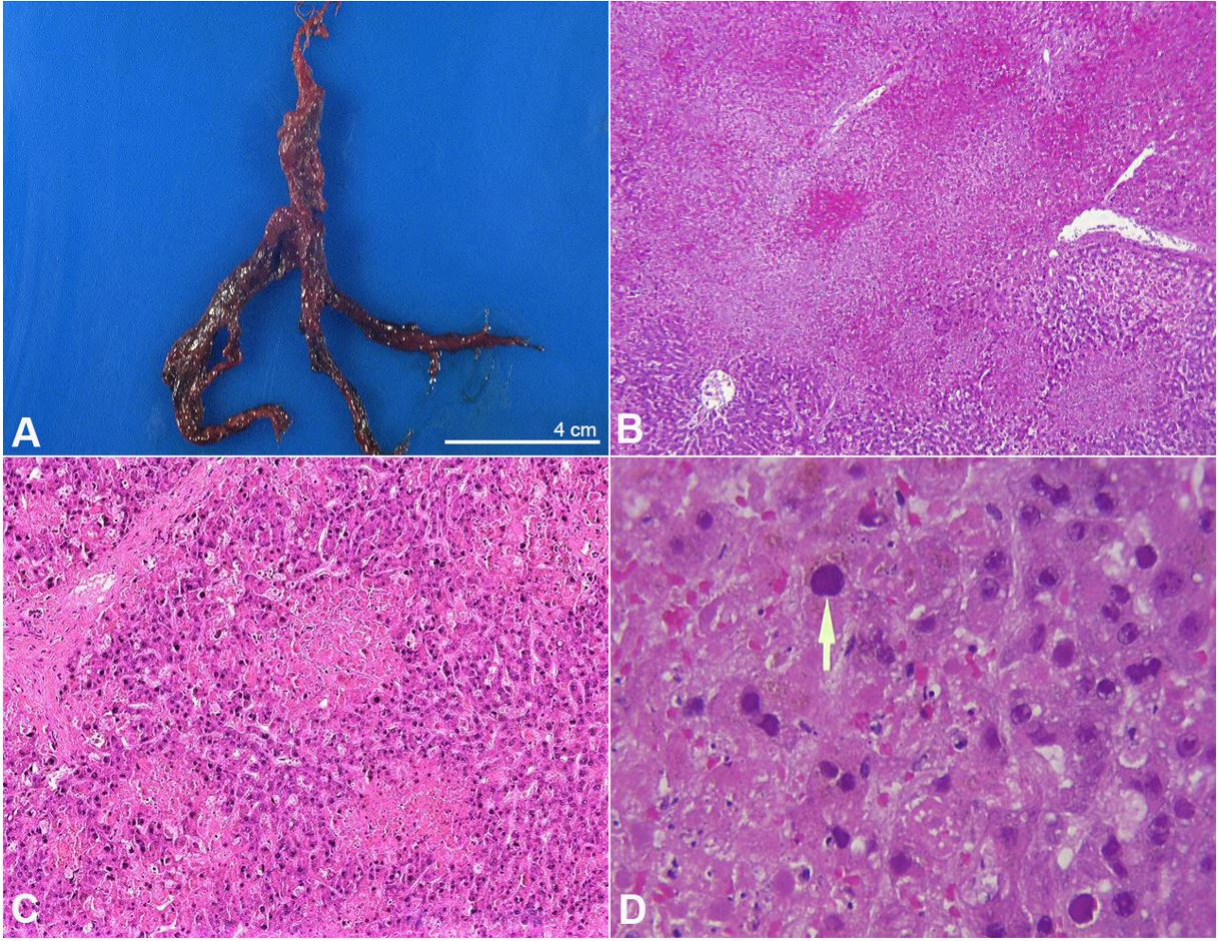
**A –** Gross view of the branching bronchial luminal blood clot, making a partial cast of bronchial tree; **B, C** and **D** photomicrographs of the liver; **B –** Area of confluent hepatic necrosis with foci of hemorrhage (H&E, 20X); **C –** Multiple small patchy areas of necrosis near a portal tract in the upper left (H&E, 50X); **D –** Hepatocytes, with large basophilic smudgy intranuclear adenovirus inclusions, one indicated by the arrow, near the edge of an area of necrosis (H&E, 400X).

Postmortem examination revealed massive bilateral acute necrotizing pneumonia with postmortem lung culture positive for heavy growth of Aspergillus fumigatus (Figure 1A, B, C, and D). In addition to these figures, a virtual (digital) slide of lung is available for viewing at the Larry Nichols Collection .^1^ It is case 005. There was severe pulmonary hemorrhage with blood clots filling much of the main bronchi (Figure 2A). Autopsy also disclosed multifocal severe acute necrotizing adenovirus hepatitis, ileitis and colitis (Figure 2B, C, and D). In addition to these figures, a virtual (digital) slide of liver is available for viewing at the Larry Nichols Collection.^1^ It is case 103. Immunostaining of liver, ileum and colon for adenovirus was positive, but immunostaining of the lungs for adenovirus was negative.

The autopsy showed severe bone marrow hypocellularity, with only rare erythroid precursors, but did not reveal graft-versus-host disease or Hodgkin disease.

## DISCUSSION

This case illustrates important aspects of Hodgkin lymphoma, aspergillosis and adenovirus infection. Hodgkin lymphoma is not a single disease, but rather a group of hematological malignancies that share many features; 80 to 90% of Hodgkin lymphomas are cured by chemotherapy.^2^ As illustrated in this case, however, there remain some patients whose Hodgkin lymphoma is refractory to chemotherapy.^3^ Autologous stem cell transplantation has become the standard of care for refractory Hodgkin lymphoma.^3^ This patient’s lymphoma was refractory to autologous transplantation, so he was given an allogeneic HLA-matched stem cell transplantation. Allogenic transplantation carries with it the risk of graft-versus-host disease. After treatment for *C. difficile* colitis in this case resulted in negative stool assay for C. difficile toxin, graft-versus-host disease was a reasonable hypothesis to explain the patient’s persistent diarrhea.

The immunosuppressive therapy given as prophylaxis against graft-versus-host disease may have played a role in the adenovirus infection disclosed by autopsy in this case. Adenovirus acquired in childhood is thought to become latent in tonsils and adenoids, and is believed to reactivate when patients later become immunocompromised.^4^ In this case, the only antemortem evidence of the intestinal and hepatic adenovirus infection revealed by the autopsy was an intestinal biopsy culture positive for the virus. This was treated with cidofovir, but cidofovir is well known for its adverse effects of renal failure and optic neuritis.^4^ When this patient developed rising creatinine after starting cidofovir, discontinuation of this known nephrotoxin, which is of limited efficacy against adenovirus, was a readily defensible choice. Continuation of cidofovir is unlikely to have halted the adenovirus Infection in this case with the ongoing need for immunosuppression.

As exemplified in this case, hematopoietic stem cell transplantation is one of the most common risk factors for adenovirus hepatitis, which is a rapidly progressive and highly lethal infection in immunocompromised patients.^5^ As illustrated in this case, the most consistent feature of adenovirus hepatitis is nonzonal hepatocyte necrosis, ranging from spotty to massive in extent, with minimal to mild associated inflammation.^5^ Associated with areas of necrosis, typically around the periphery, are hepatocytes with characteristic smudgy intranuclear inclusions, which are most often basophilic on H&E stain.^6^ If the inclusions are recognized on H&E stain, the diagnosis of adenovirus infection can be confirmed by immunostain.

The diagnosis of adenovirus hepatitis was correlated with a high De Ritis ratio of AST/ALT in a study of clinicopathologic correlations.^5^ Viral etiology of acute hepatitis is typically associated with a low AST/ALT (De Ritis) ratio, while alcoholic hepatitis is associated with a high AST/ALT (De Ritis) ratio.^7^ The AST/ALT ratio in normal liver is 2.5 and one explanation for the low ratios, less than 1.0, often seen in acute viral hepatitis, is that these are tested in the resolving phase when the longer serum half-life of ALT (36 hours), compared to AST (18 hours), comes into play. With worsening acute viral hepatitis, the ratio is more often between 1.5 and 2.0, and with fulminant acute viral hepatitis, over 2.0.^7^ This is, however, primarily from analysis of hepatitis A, B, C, D and E due to hepatotropic viruses. Adenovirus infects other organs, too. The explanation offered for the high AST/ALT ratio in the study of clinicopathologic correlations of adenovirus hepatitis is the release of AST from organs besides liver.^5^ In fulminant, especially fatal, hepatitis, patients go into shock and AST is released from all sorts of extrahepatic tissues due to ischemia and necrosis. In the case of this report, the AST/ALT (De Ritis) rose from 1.4 on admission to 2.1 on hospital day 5, 2.6 on day 6 and 3.9 on day 7, when the patient died. It is noteworthy that he did not become hypotensive until a few hours antemortem, so ischemic necrosis of extrahepatic tissue is not likely the best explanation of the high AST/ALT ratio in this case. The autopsy provides an alternative explanation; it showed severe acute necrotizing ileitis and colitis, an extrahepatic source of AST. Of course, worsening hepatitis also no doubt played a role. The advent of specific effective therapy for adenovirus infection will make all the ways of suspecting and making the specific diagnosis important.

The patient in this case also had invasive pulmonary aspergillosis, which begins with the inhalation of Aspergillus spores.^8^ These spores are ubiquitous in both indoor air and outdoor air. They get into the air from household dust, soil, food, water, plants, flowers, decomposing plants, and building materials.^8^ In a study of the air inside and outside the homes of patients with invasive pulmonary aspergillosis and controls, every single home had Aspergillus fumigatus in the air.^9^ In normal hosts, the small, 2 to 3 micron Aspergillus spores are phagocytosed by alveolar macrophages and destroyed with reactive oxygen species in lysosomes.^10^ In immunocompromised individuals, spores can evade this defense and germinate into fungal hyphae, which invade tissue of the lower respiratory tract.

Invasive aspergillosis of the lower respiratory tract typically causes acute necrotizing pneumonia, with angioinvasion and thrombosis, often with hemorrhage, infarcts and cavities.^11^ All of these features were evident microscopically in the case of this report. Aspergillus hyphae in invasive pulmonary infection are typically regular septate hyphae with progressive branching at acute angles, in a radiating starburst pattern, best appreciated on Grocott methenamine silver staining.^11^ In patients who have received antifungal therapy, the hyphae may become irregular, with ballooning of some segments, and smaller numbers of hyphae, in haphazard arrangements. All of these features were evident in this case. Also evident was an atypical feature: the branching luminal blood clots, making partial casts of the bronchial tree. This correlates with the hemoptysis the patient began having on hospital day 2, starting with blood-tinged sputum.

The symptoms of invasive pulmonary aspergillosis include fever, cough, dyspnea, and hemoptysis.^12^ The most common symptom of invasive pulmonary aspergillosis is fever.^12^ The patient of this report had fever as a presenting symptom on admission, but in the hospital, he had only a low grade fever of 38.2 °C and that only on the second day. A great majority of patients with invasive pulmonary aspergillosis have cough and dyspnea as symptoms of it.^12^ This patient had both cough and dyspnea. Hemoptysis is a rare symptom of invasive pulmonary aspergillosis, but this patient had it. The symptoms are all nonspecific and aspergillosis is not the most common cause of any of them, so it is difficult to suspect. Invasive pulmonary aspergillosis should be suspected when symptoms or signs occur in patients at risk. Neutropenia is the major risk factor, especially prolonged neutropenia.^8^ This makes sense because innate immunity is the major defense against aspergillosis. This patient had progressive neutropenia, with a fall in his WBC count from 1,900/mm^3^ on admission to 200/mm^3^ on the day he died. Beyond neutropenia, among adults, the highest risk groups are those with lung transplantation, induction chemotherapy for acute myeloid leukemia, and allogeneic hematopoietic stem cell transplantation, all with around a 25% incidence.^8^ This patient had hematopoietic stem cell transplantation. Other risk groups include patients receiving corticosteroid therapy and those receiving immunosuppressive molecular biologic agents for autoimmune diseases.

Even when invasive pulmonary aspergillosis is suspected, it is difficult to diagnose.^8^ ^,^ ^12^ Definitive diagnosis requires both identification of invasion in tissue and identification of the species in culture.^13^ Positive culture alone can represent merely colonization or even laboratory contamination.^8^ Tissue invasion by septate fungal hyphae with acute angle branching can represent infection by Scedosporium, Fusarium or other species, often resistant to the antifungal agents effective against Aspergillus species.^14^ The yield of culture and transbronchial biopsy is low, as illustrated by the false negative biopsy in this case. Bronchoalveolar lavage cytology was positive in this case, but this also has a low yield overall, lower than culture.^15^ Serum galactomannan was also positive in this case. Galactomannan is a polysaccharide component of fungal cell wall that can be assayed in blood or bronchoalveolar lavage fluid. It is more sensitive than culture for the detection of Aspergillus species.^8^ ^,^ ^13^ Serum galactomannan is positive in 60 to 80% of patients with aspergillosis, neutropenia and hematological malignancy, but <50% in non-neutropenic patients.^8^ The specificity of serum galactomannan is 85 to 90%, but other mold and dimorphic fungal species, such as Fusarium, Paecilomyces, Penicillium, Acremonium, Alternaria, Wangiella, Histoplasma and Blastomyces can all have a positive serum galactomannan.^8^ ^,^ ^13^

Even when invasive pulmonary aspergillosis is suspected and then diagnosed, it is difficult to treat.^8^ Surgery in addition to medical therapy may be required for patients with severe hemoptysis.^13^ This patient’s invasive pulmonary aspergillosis progressed over his 7-day hospitalization, despite caspofungin therapy since admission, adding voriconazole on day 3 and adding posaconazole on day 6. Caspofungin is not as effective for invasive pulmonary aspergillosis as voriconazole or posaconazole.^8^ This antifungal therapy, however, was empiric because the only laboratory tests positive for fungal infection (bronchoalveolar lavage cytology and blood galactomannan) did not return until after the patient’s death.

As you read the clinical history of this case, if you imagine yourself as the patient’s clinician, you should be impressed at the failure of each successive treatment, the complications of successive treatments, and finally the force of the two fatal opportunistic infections revealed by the autopsy. This case illustrates some of the features characteristic of adenovirus hepatitis and invasive pulmonary aspergillosis, but what it illustrates perhaps even better are the limits of modern medicine. Physicians diagnose diseases and understand their pathophysiology, which were mysteries just a few decades ago, like the thrombotic thrombocytopenic purpura in this case. They give sophisticated treatments that were imaginary basic science fiction for doctors only one or two generations ago like plasmapheresis and the two types of stem cell transplantation in this case. It is humbling to see all the numerous powerful life-saving measures of modern medicine employed in this patient’s care evaded by the grim reaper (death).

## CONCLUSION

This is the report of a case of Hodgkin lymphoma refractory to chemotherapy that was treated with allogeneic hematopoietic stem cell transplantation complicated by adenovirus enteritis and hepatitis, together with invasive pulmonary aspergillosis. The adenovirus infection and aspergillosis were diagnosed only at autopsy. The patient presented with dyspnea and fever unresponsive to antibacterial therapy for respiratory pathogens not already covered by the prophylactic antibiotics he was receiving. Computed tomography of the chest showed a right perihilar infiltrate and a separate right upper lobe infiltrate. The patient also had diarrhea and elevated liver enzyme levels. He suffered a 7-day progressive downhill course despite broad-spectrum antibiotic therapy, ultimately including three antifungal medications. This case illustrates many of the clinical and pathologic features of aspergillosis and adenovirus hepatitis. It shows the value of autopsy in revealing diagnoses. Perhaps more than anything else, however, it illustrates the limits of modern medicine, which should serve as a mental spur in our efforts to advance medical science, to try to defeat the numerous demons of disease, who seem to keep outwitting us.
